# Parallel and Visual Detections of ASFV by CRISPR-Cas12a and CRISPR-Cas13a Systems Targeting the Viral S273R Gene

**DOI:** 10.3390/ani15131902

**Published:** 2025-06-27

**Authors:** Hongjian Han, Desheng Zhang, Weilin Hao, Anjing Liu, Nengwen Xia, Meng Cui, Jia Luo, Sen Jiang, Wanglong Zheng, Nanhua Chen, Jinguo Gu, Jianfa Bai, Jianzhong Zhu

**Affiliations:** 1 College of Veterinary Medicine, Yangzhou University, Yangzhou 225009, China; mz120231693@stu.yzu.edu.cn (H.H.);; 2 Joint International Research Laboratory of Agriculture and Agri-Product Safety, Yangzhou University, Yangzhou 225009, China; 3 Comparative Medicine Research Institute, Yangzhou University, Yangzhou 225009, China; 4 Jiangsu Co-Innovation Center for Prevention and Control of Important Animal Infectious Diseases and Zoonoses, Yangzhou University, Yangzhou 225009, China; 5 Kangnongmu Animal Husbandry Ecological Farm, Yancheng 224025, China; 6 Kansas State Veterinary Diagnostic Laboratory, Kansas State University, Manhattan, KS 66506, USA

**Keywords:** ASFV, detection, CRISPR-Cas12a, CRISPR-Cas13a, S273R gene

## Abstract

African swine fever virus (ASFV) causes a highly contagious and lethal hemorrhagic disease and significantly threatens the pig industry. There is no commercially effective vaccine available currently, making the detection of ASFV critical for control and prevention. Previously, we established the CRISPR-LbCas12a and LwCRSIRP-Cas13a visual detections of ASFV, separately, targeting the structural p17 gene D117L. In this study, we performed the parallel detections of ASFV based on the conserved viral protease gene S273R using CRISPR-LbCas12a and CRISPR-LbuCas13a systems. Our results showed that both systems are able to specifically detect ASFV as low as two copies of the S273R gene, and effectively detect clinical samples with minimal DNA purification. The work promotes CRISPR-Cas systems for the application of on-site detection in the field.

## Simple Summary

African swine fever virus causes a highly contagious and lethal disease, and significantly threatens the pig industry worldwide. There is no commercially effective vaccine available, making the detection of African swine fever virus the key for control and prevention. Clustered regularly interspaced short palindromic repeats-Caspase is a newly developed state-of-the-art technology for detection. Here, we develop the parallel detections of African swine fever virus based on the conserved viral protease gene S273R using Caspase 12a and Caspase 13a systems. The parallel detections are highly consistent with quantitative polymerase chain reaction detection, and effectively detect clinical samples without deoxyribonucleic acid purification, significantly advancing the on-site detection in the field.

## 1. Introduction

African swine fever virus (ASFV), a double-stranded DNA virus of the family *Asfarviridae*, causes African swine fever (ASF), a highly contagious and lethal hemorrhagic disease affecting pigs of all ages and breeds, with mortality rates approaching 100% [1]. Since its first outbreak in Kenya in 1920, ASFV has rapidly spread across Africa and Eurasia [1]. In 2018, its emergence in China resulted in substantial economic losses to the swine industry [2,3]. Clinical manifestations of ASF include fever, cyanosis, and systemic hemorrhaging in organs [4,5]. Currently, no effective vaccines or therapeutics are available, underscoring the urgent need for rapid, sensitive, cost-effective diagnostic methods to curb ASFV transmission [4,6]. Existing techniques, such as conventional PCR, quantitative PCR, ELISA, and fluorescent antibody assays, often require skilled personnel and sophisticated equipment, limiting their utility for on-site detection [7,8].

In recent years, CRISPR-Cas-based detection systems have gained prominence due to their high specificity, sensitivity, simplicity, and rapidity [9]. CRISPR systems utilize guide RNA (gRNA) to activate Cas nuclease activity [10,11,12]. For instance, Cas12a and Cas13a recognize protospacer sequences in target nucleic acids, forming a crRNA-target duplex that triggers both sequence-specific (cis-cleavage) and nonspecific (trans-cleavage) enzymatic activities [10,11,12]. The latter enables indiscriminate degradation of single-stranded DNA (ssDNA) or RNA (ssRNA) in the reaction environment [10,11,12]. To enhance clinical applicability, recombinase polymerase amplification (RPA) is usually incorporated to rapidly amplify the template DNA under ambient conditions, which utilizes recombinant enzymes, strand-displacing enzymes, and single-strand binding proteins [13]. RPA has high sensitivity and speed, and good compatibility with CRISPR-Cas systems, enabling the integration of RPA, CRISPR-Cas systems, and lateral flow strip (LFS) technologies into a rapid, portable, and visually interpretable diagnostic platform [14,15].

ASFV possesses a genome of 170–194 kb, encoding approximately 200 viral proteins [16]. Among these, pS273R is a SUMO-1 cysteine protease critical for the maturation of polyprotein pp220 and pp62 into core–shell proteins (p5, p34, p14, p37, p150, p15, p35, and p8) [17,18]. The pS273R protease comprises 271 amino acids organized into two domains: a conserved “core domain” and an N-terminal “arm domain” unique to ASFV, which is essential for maintaining protease activity [19]. Functionally, pS273R inhibits cGAS-STING-mediated IFN response via different mechanisms [20,21], suppresses the formation of inflammasomes [22] and stress granules [23], as well as STAT2 expression [24], to evade the host antiviral immunity.

In this study, we targeted the S273R gene to develop an ASFV detection method by integrating RPA amplification, CRISPR-LbCas12a/LbuCas13a-mediated cleavage, and LFS-based visualization. Custom-designed RPA primers, crRNAs, fluorescent probes, and biotinylated probes were synthesized, the reaction conditions were systematically optimized, and the detection sensitivity and specificity were rigorously evaluated. Clinical samples were analyzed for parallel RPA-CRISRP-LbCas12a/LbuCas13a detections using two DNA extraction approaches: (1) column-based method and (2) lysis-based DNA extraction, with results validated against qPCR. Our findings demonstrated a high detection concordance between the RPA-CRISPR-LFS platforms and qPCR.

## 2. Materials and Methods

### 2.1. Experimental Reagents

The plasmid p2CT-His-MBP-Lbu_C2c2_WT (Cat# P1104) encoding LbuCas13a was purchased from Miaoling Biotechnology Co., Ltd. (Wuhan, China). The recombinant polymerase amplification (RPA) kit (TwistAmp Basic, Cat #TABAS03KIT) was obtained from TwistDx Limited (Maidenhead, UK). T7 High Yield RNA Transcription Kit (Cat# TR101-01), FastPure Gel DNA Extraction Mini Kit (Cat# DC301-01), and FastPure Plasmid Mini Kit-BOX 2 (Cat# DC201-01) were all from Vazyme Biotech (Nanjing, China). Spin Column RNA Cleanup Concentration Kit (Cat# B518688-0050), and DNA-EZ Reagents V All-DNA-Out (Cat# B642315-0010) were bought from Sangon Biotech (Shanghai, China). HiPure Tissue DNA Mini Kit (Cat# D3123-02) was purchased from Magen Biotechnology (Guangzhou, China). His-tag Protein Purification Kit (Reductant & Chelator-resistant) (Cat# P2226) and TEV protease (Cat# P2307) were obtained from Beyotime Biotechnology (Shanghai, China).

### 2.2. Expression and Purification of LbCas12a Protein

The purification of LbCas12a was performed as we described previously [25]. Briefly, a single colony of pET28a-Cas12a plasmid transformed BL21 *E. coli* was inoculated into 5 mL LB broth and cultured at 37 °C with shaking at 220 rpm for 10–14 h. The culture was then scaled up to 500 mL LB broth and grew until OD_600_ reached 0.6–0.8. Protein expression was induced with 1 mM IPTG at 16 °C for 16 h under continuous shaking at 220 rpm. Bacterial cells were harvested by centrifugation and lysed via sonication. The lysate was clarified by centrifugation, and the LbCas12a in the supernatant was purified using Ni-NTA affinity. The eluted protein was dialyzed thrice against storage buffer (50 mM Tris-HCl, 600 mM NaCl, 5% glycerol, 2 mM DTT, pH 7.5) at 4 °C using a shaking incubator, with buffer replaced every 2 h.

### 2.3. Expression and Purification of LbuCas13a Protein

The commercial p2CT-His-MBP-Lbu_C2c2_WT plasmid was transformed into BL21 competent cells. A single colony was inoculated into 5 mL LB broth and cultured at 37 °C with shaking (220 rpm) for 12–16 h. The culture was expanded to 500 mL LB broth and grown until OD_600_ reached 0.4–0.8. Protein expression was induced with 0.5 mM IPTG at 16 °C for 20 h under shaking (220 rpm). Bacterial cells were harvested and lysed via sonication. The His-MBP-LbuCas13a fusion protein was purified from the lysate supernatant via Ni-NTA affinity column from the bacterial lysate supernatant and dialyzed against buffer (50 mM Tris-HCl, 250 mM KCl, 5% glycerol, 1 mM TCEP, pH 7.5) thrice at 4 °C. The MBP tag was cleaved by incubating the purified fusion protein with TEV protease at 4 °C for 10–16 h. The cleavage mixture was reapplied to a Ni-NTA column, and the flow-through containing tag-free LbuCas13a was collected. The purified LbuCas13a was identified by SDS-PAGE and subsequent Coomassie blue staining.

### 2.4. Design and Preparation of LbCas12a-crRNA and Template DNA Targeting the ASFV S273R Gene

The conserved S273R gene of ASFV was selected as the detection target, and the crRNAs were designed. Each crRNA is comprised of the T7 promoter sequence (TAATACGACTCACTATAG) + Cas12a scaffold sequence (AATTTCTACTAAGTGTAGAT) + a 20–30 bp target-specific sequence downstream of the protospacer adjacent motif (PAM) sequence (TTTN). Complementary single-stranded DNA (ssDNA) templates encoding crRNAs were synthesized, annealed, and transcribed in vitro using the T7 High Yield RNA Transcription Kit. The transcribed crRNAs were purified using the Spin Column RNA Cleanup Concentration Kit.

For DNA template amplified by recombinase polymerase amplification (RPA), primers (30–35 bp each) targeting the conserved regions of the ASFV S273R gene were designed to generate a ~200 bp amplicon (Table S1). Three crRNAs (crRNA1–3), two pairs of RPA primers, and one pair of PCR primers were designed (Table S1). The cleavage activities mediated by crRNAs in the CRISPR-Cas12a reaction were evaluated by blue light (450 nm), ultraviolet (UV) light (320 nm), and agarose gel electrophoresis.

### 2.5. Design and Preparation of LbuCas13a-crRNA and Template RNA Targeting the ASFV S273R Gene

The crRNAs for LbuCas13a were designed with the T7 promoter sequence, LbuCas13a scaffold sequence (GACCACCCCAAAAAUGAAGGGGACUAAAAC), and a 20–30 bp target sequence downstream of the protospacer flanking sequence (PFS, non-G nucleotide). Complementary ssDNA templates were synthesized, annealed, transcribed in vitro, and the transcribed RNAs were purified as described above.

To generate an RNA template for LbuCas13a detection, the forward RPA primer was modified to include a T7 promoter sequence (Table S1). RPA-amplified DNA products were transcribed into RNA using the T7 High Yield RNA Transcription Kit. Two crRNAs (crRNA1–2), three pairs of RPA primers, and one pair of PCR primers were designed (Table S1), and their cleavage activities were validated by blue-light and UV-light detection, and agarose gel electrophoresis.

### 2.6. RPA-CRISPR-LbCas12a/LbuCas13a Detection Systems

The LbCas12a-crRNA complex recognizes target template DNA (RPA-amplified product) and activates collateral cleavage of the ssDNA probe. The reaction mixture contained: 44 μL detection buffer (20 mM Tris-HCl, 100 mM KCl, 6 mM MgCl2, 1 mM DTT, 5% glycerol, 50 μg/mL heparin, pH 7.5); 1 μL crRNA (500 ng/μL); 2 μL LbCas12a protein (250 ng/μL); 2 μL template DNA; 1 μL 5′-FAM-BHQ-3′ ssDNA probe (8 μM, Table S1), in total 50 μL. The reaction was incubated at 37 °C for 30–60 min in a water bath. DNA cleavage activity was visualized under blue light (450 nm) or UV light (320 nm).

The LbuCas13a-crRNA complex binds to the template of transcribed RNA (transcribed product of the RPA amplicon) and induces collateral cleavage of the ssRNA probe. The reaction mixture contained: 43.5 μL detection buffer (20 mM Tris-HCl, 100 mM KCl, 6 mM MgCl_2_, 1 mM TCEP, 5% glycerol, pH 7.5); 1 μL crRNA (250 ng/μL); 1 μL LbuCas13a protein (250 ng/μL); 2 μL transcribed RNA template; 1 μL 5′-FAM-BHQ-3′ ssRNA probe (2 μM, Table S1); 0.5 μL RNase inhibitor (40 U/μL), in total 50 μL. The reaction was performed at 37 °C for 15–30 min. RNA cleavage activity was detected under blue light (450 nm) or ultraviolet (UV) light (320 nm).

### 2.7. RPA-CRISPR-LbCas12a/LbuCas13a Lateral Flow Strip (LFS) Detection Systems

The 5′-FAM-biotin-3′ dual-labeled ssDNA and ssRNA probes were prepared and substituted for 5′-FAM-BHQ-3′ probes (Table S1), while maintaining other optimal CRISPR-Cas reaction conditions mentioned above. The sample application zone (arrow end) of the LFS was immersed in the reaction liquid for 5–10 min. In both LbCas12a-LFS and LbuCas13a-LFS detection systems, the following two results may occur: (a) Negative result, the intact FAM-biotin probe binds to gold nanoparticle-conjugated anti-FAM antibody, forming a complex that migrates along the strip. The biotin moiety is captured by streptavidin immobilized on the control line (C line), generating a visible gold band at the C line. (b) Positive result, the FAM-biotin probes are cleaved by activated LbCas12a or LbuCas13a into the FAM fragment and biotin fragment. Whereas the biotin fragment is captured at the C line, the FAM fragment binds to gold-conjugated anti-FAM antibody, and the complex migrates past the C line, continues to flow, and is captured by anti-rabbit IgG immobilized on the test line (T line), producing a distinct gold band at the T line. If a large amount of the ASFV target is detected, the complete probe cleavage avoids C line retention, with only T gold signal appearance.

### 2.8. Processing and Detection of Clinical Specimens by CRISPR-Cas Systems

The genomic DNA (gDNA) was extracted with the following two methods: (a) Column-based genomic DNA extraction. The tissue samples were finely minced and subjected to enzymatic digestion using proteinase K (20 mg/mL) and RNase A (10 μg/mL) at 70 °C for 10 min. Absolute ethanol was added to the lysate, followed by vortex mixing (10 s). The mixture was loaded onto a HiPure Tissue DNA Mini Column (Cat# DNC27-01, Magen Biotech, Guangzhou, China) for silica-membrane-based purification. The column was washed twice with wash buffer and gDNA was eluted using the preheated (70 °C) elution buffer, followed by RPA amplification. (b) Universal one-step DNA extraction [26]. A rapid DNA extraction buffer (DNA-EZ Reagents V All-DNA-Out, RPA compatible) was used, and ~2 mg tissue samples were homogenized and added to the 50 μL buffer. The mixture was incubated at 95 °C for 5 min, vortexed briefly, and centrifuged (12,000× *g*, 1 min), with the supernatant containing crude DNA directly used for RPA. The RPA products were used for subsequent CRISPR-Cas reactions.

## 3. Results

### 3.1. Development of CRISPR-LbCas12a/LbuCas13a Systems Detecting the ASFV S273R Gene

**Figure 1:**
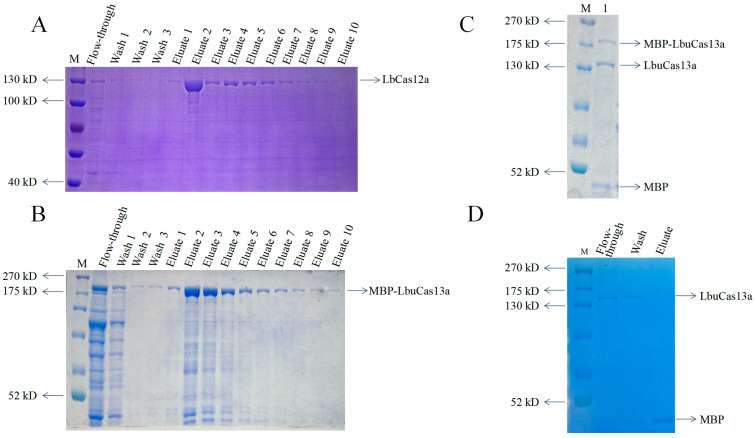
Purification of LbCas12a and LbuCas13a proteins: (**A**) Purification of His-tagged LbCas12a protein by Ni-NTA affinity chromatography. Ten rounds of elution were performed to obtain high-purity LbCas12a protein. (**B**) Purification of His-tagged MBP-LbuCas13a fusion protein via Ni-NTA affinity. Ten rounds of elution yielded the MBP-LbuCas13a fusion protein. (**C**) TEV protease cleavage of MBP-LbuCas13a fusion protein in lane 1. (**D**) Further purification of TEV-cleaved products using Ni-NTA affinity to remove the His-MBP tag and un-cleaved fusion protein. M, protein marker, with the protein bands of different sizes indicated. The LbCas12a, MBP-LbuCas13a, LbuCas13a, and MBP are referred to by arrows.

The eluted LbCas12a showed as 130 kD protein bands, as expected (Figure 1A). As for LbuCas13a, the eluted MBP-LbuCas13a showed as >180 kD protein bands (Figure 1B). TEV protease enabled MBP to be cut from LbuCas13a (Figure 1C), and purified LbuCas13a showed as 150 kD protein bands (Figure 1D). The purified LbCas12a and LbuCas13a proteins were used for CRISPR-Cas12a and CRISPR-Cas13a detections, respectively.

**Figure 2:**
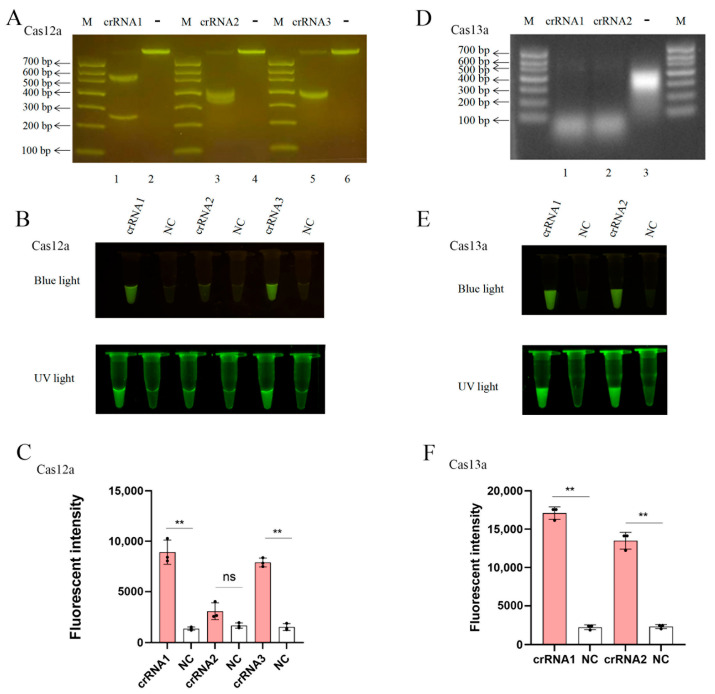
Verification of crRNA-mediated CRISPR-Cas12a/Cas13a nuclease activity: (**A**) Agarose gel electrophoresis of S273R PCR amplicon of target DNA cleaved by LbCas12a crRNAs 1-3. (**B**) Detection of CRISPR-LbCas12a trans-cleavage activity under blue light (450 nm) and ultraviolet (UV) light (320 nm). (**C**) Quantitative analysis of blue-light signals in panels B using Image J software (version 1.54k). (**D**) Agarose gel analysis S273R target RNA transcribed from a PCR amplicon and cleaved by LbuCas13a crRNAs 1–2. (**E**) Detection of CRISPR-LbuCas13a trans-cleavage activity under blue light and UV light. (**F**) Quantitative analysis of blue-light signals in E using Image J software. M, DNA marker with the sizes of DNA bands marked. NC, negative controls without crRNA. ** *p* < 0.01 and ns means not statistically significant by Student *t* test.

We successfully established CRISPR-LbCas12a and CRISPR-LbuCas13a detection systems targeting the conserved ASFV S273R gene. For the LbCas12a system, all three designed crRNAs (crRNAs 1–3) mediated the collateral cleavage activity. In agarose electrophoresis, the 822 bp DNA of S273R PCR amplicon as the template was cleaved into two fragments; crRNA1 generated 537 bp and 285 bp fragments, crRNA2 produced 390 bp and 432 bp fragments, and crRNA3 yielded 410 bp and 412 bp fragments (Figure 2A). Among three crRNAs, both crRNA1 and crRNA3 exhibited significant cleavage efficiency on ssDNA probe, under both blue light and UV light (Figure 2B,C). In general, crRNA1 was slightly better than crRNA3 in mediating Cas12a cutting efficiency. For the LbuCas13a system, two designed crRNAs were evaluated for the cis-cleavage activity on template RNA and trans-cleavage activity on ssRNA probe. Both crRNAs mediated cleavage of template RNA transcribed from a PCR amplicon in agarose electrophoresis (Figure 2D), as well as probe cleavage, under both blue and UV light (Figure 2E,F), with crRNA1 showing higher activity. Therefore, the crRNA1 of LbCas12a and crRNA1 of LbuCas13a were chosen for subsequent experiments.

### 3.2. Optimization of CRISPR-LbCas12a/LbuCas13a Detection Systems

The probe concentrations, Cas protein concentrations, and crRNA concentrations for both CRISPR-LbCas12a and CRISPR-LbuCas13a systems were systematically optimized as follows.

**Figure 3:**
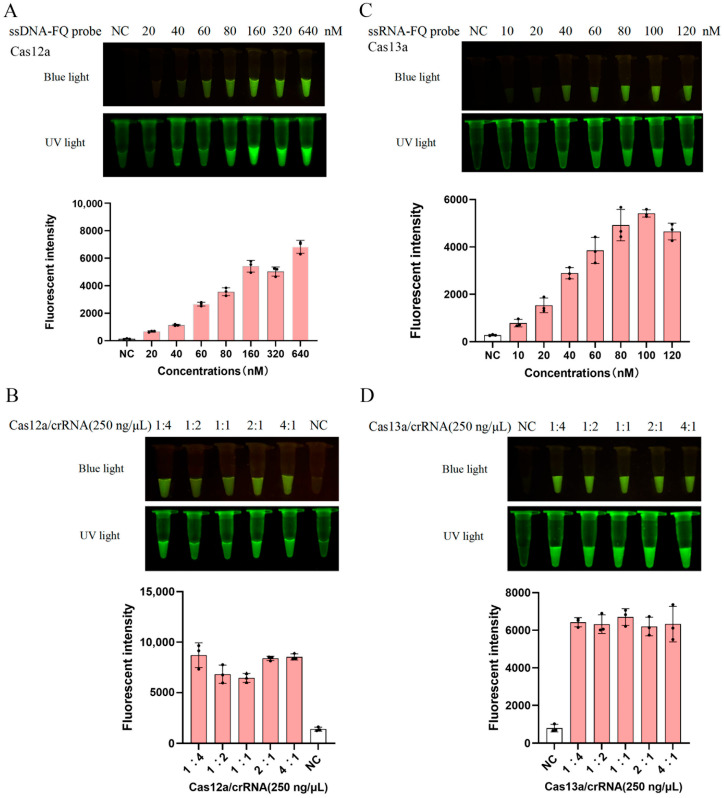
Optimization of CRISPR-LbCas12a/LbuCas13a reaction conditions: (**A**) Detection of trans-cleavage activity of CRISPR-LbCas12a at gradient probe concentrations (20–640 nM) under blue light and UV light, with the quantification of blue-light signals presented. (**B**) Evaluation of cleavage efficiency at varying LbCas12-to-crRNA ratios (1:4, 1:2, 1:1, 2:1, 4:1) under blue light and UV light, with the quantification of blue-light signals presented. (**C**) Detection of trans-cleavage activity of CRISPR-LbuCas13a at gradient probe concentrations (10–120 nM) under blue light and ultraviolet light, with the quantification of blue-light signals presented. (**D**) Evaluation of cleavage efficiency at varying LbuCas13a-to-crRNA ratios (1:4, 1:2, 1:1, 2:1, 4:1), under blue light and UV light, with the quantification of blue-light signals presented.

For the CRISPR-LbCas12a system, the ssDNA probe was tested at concentrations of 20, 40, 80, 160, 320, and 640 nM, and a probe concentration range of 160–320 nM was selected based on optimal signal-to-noise ratios (Figure 3A). The initial concentrations of LbCas12a protein and selected crRNA 1 were both set to 250 ng/μL, and reactions were performed at varying ratios (1:4, 1:2, 1:1, 2:1, and 4:1) (Figure 3B). The results demonstrated that a 2:1 ratio (500 ng/μL LbCas12a, 250 ng/μL crRNA 1) achieved the highest cleavage efficiency (Figure 3B).

For the CRISPR-LbuCas13a system, the ssRNA probe was evaluated at concentrations of 10, 20, 40, 60, 80, and 120 nM, and the probe concentration range of 80–100 nM was determined to yield optimal performance (Figure 3C). The initial concentrations of LbuCas13a protein and selected crRNA 1 were both set to 250 ng/μL, and reactions were conducted across ratios of 1:4, 1:2, 1:1, 2:1, and 4:1. The results indicated that a 1:1 ratio (250 ng/μL LbuCas13a, 250 ng/μL crRNA 1) provided the best detection outcomes (Figure 3D).

### 3.3. Establishment of RPA-CRISPR-LbCas12a/LbuCas13a Lateral Flow Strip (LFS) Detection Methods

**Figure 4:**
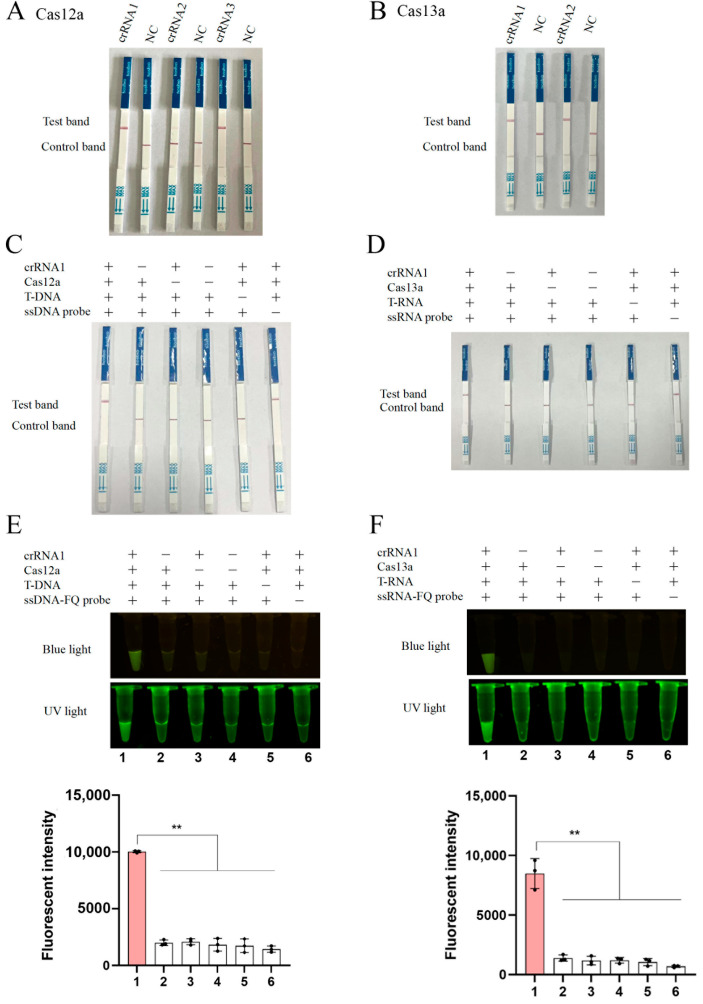
Verification of CRISPR-LbCas12a/LbuCas13a lateral flow strip (LFS) detections: (**A**,**B**) LFS detections mediated by three LbCas12a crRNAs 1-3 (**A**) and two LbuCas13a crRNAs 1-2 (**B**). (**C**,**D**) Assay integrity testing. The omission of individual components (crRNA, Cas protein, template, or probe) abolished target-specific signals, while omission of the probe generated false-positive signals due to non-specific anti-FAM-gold retention at T lines. (**E**,**F**) Comparative fluorescence detection results were obtained, corresponding to panels (**C**) and (**D**), respectively, with the quantifications of blue-light signals presented. ** *p* < 0.01 by Student *t* test.

To facilitate ASFV detection of clinical samples, we integrated the CRISPR-LbCas12a/LbuCas13a and colloidal gold LFS to establish a rapid diagnostic platform. The 5′-FAM-biotin-3′ dual-labeled ssDNA and ssRNA probes were designed for signal generation. If the ASFV target is absent, the intact FAM-biotin probe binds to gold particle-conjugated anti-FAM antibody, forming a complex that migrates along the strip. The biotin moiety of the complex will be captured by streptavidin immobilized on the C line, producing a gold band exclusively at the C line. If the ASFV target is present, the cleavage of the FAM-biotin probe occurs. A fraction of intact probe generates a C line signal, while the cleaved FAM gold complex passes the C line, migrates to the T line, and will be captured by anti-rabbit IgG, yielding dual gold bands (C and T lines). If a large amount of the ASFV target is detected, the complete probe cleavage prevents C line retention, with only T gold signal appearance. The LbCas12a crRNAs 1 and 3, and LbuCas13a crRNAs 1 and 2 exhibited obvious cleavage activity against the FAM-biotin probes in colloidal gold lateral flow strip assays (Figure 4A,B). The assays required all four components, including crRNAs, LbCas12a/LbuCas13a proteins, target templates, and FAM-biotin probes, to function correctly, and omission of any component abolished specific detection signals (Figure 4C,D). Noticeably, the absence of FAM-biotin probes resulted in non-specific anti-FAM-gold retention at T lines, thus generating the false-positive signals. Generally, the LFS results were fully consistent with those of fluorescence-based detections (Figure 4E,F).

### 3.4. Sensitivity and Specificity of the RPA-CRISPR-LbCas12a/LbuCas13a Detection Methods

**Figure 5:**
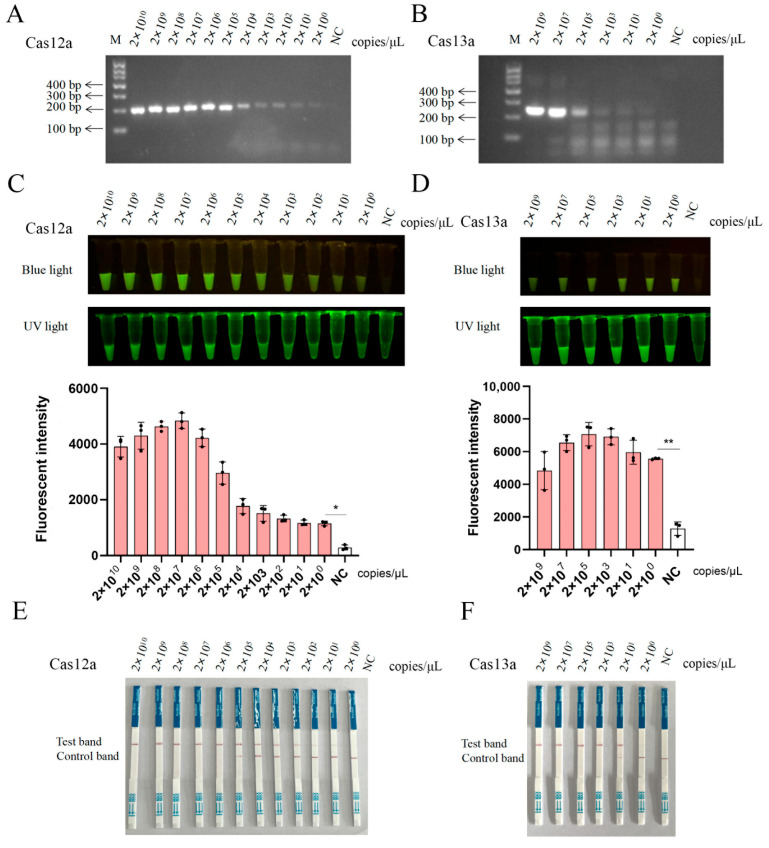
Sensitivity of CRISPR-LbCas12a/LbuCas13a detection assays: (**A**,**B**) Agarose gel electrophoresis of two RPA-amplified products from serially diluted pCAGGS-S273R plasmid (2 × 10^10^–2 × 10^0^ copies/μL). (**C**,**D**) RPA-amplified products from (**A**,**B**) were analyzed using the CRISPR-LbCas12a system (**C**) and CRISPR-LbuCas13a system (**D**), respectively, with results visualized under blue light and UV light to confirm target-specific cleavage activity. The quantifications of blue-light signals were presented and shown below. (**E**,**F**) Lateral flow strip (LFS) validation of the detection sensitivity of CRISRP-LbCas12a (**E**) and CRISRP-LbuCas13a (**F**), showing concordance with fluorescence results in (**C**,**D**). NC, negative controls without plasmid templates. * *p* < 0.05 and ** *p* < 0.01 by Student *t* test.

We further integrated RPA into the CRISPR-LbCas12a/LbuCas13a-LFS platforms to achieve sensitive and rapid detections. To assess the sensitivity of the RPA-CRISPR-LbCas12a/LbuCas13a detection systems, the pCAGGS-S273R plasmid was serially diluted from 2 × 10^10^ copies/μL to 2 × 10^0^ copies/μL, followed by RPA with the best primers (Table S1) using the diluted plasmids as templates (Figure 5A,B). One RPA-amplified DNA products (Figure 5A) were directly subjected to the crRNA1-mediated CRISPR-LbCas12a assay, whereas another RPA products (Figure 5B) were transcribed into RNA in vitro using T7 RNA polymerase for crRNA1-mediated CRISPR-LbuCas13a detection. Both CRISPR assays achieved a detection limit of 2 × 10^0^ copies/μL, as confirmed by fluorescence signals (Figure 5C,D) and LFS analysis (Figure 5E,F).

**Figure 6:**
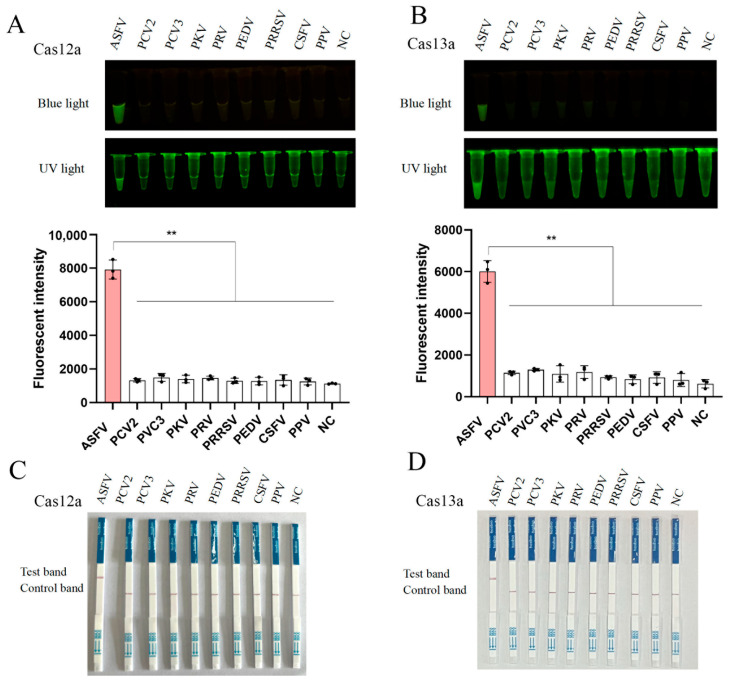
Specificity of CRISPR-LbCas12a/LbuCas13a detection assays: (**A**,**B**) Fluorescence-based specificity testing against ASFV and eight other porcine viruses (PCV-2, PCV-3, PKV, PRV, PEDV, PRRSV, CSFV, PPV). Signals were observed exclusively for ASFV in both CRISRP-LbCas12a detection (**A**) and CRISPR-LbuCas13a detection (**B**). The quantifications of blue-light signals were presented and shown below. (**C**,**D**) LFS-based specificity testing against ASFV and eight other porcine viruses. Signals were observed exclusively for ASFV in both CRISPR-LbCas12a-LFS detection (**A**) and CRISPR-LbuCas13a-LFS detection (**B**), with no cross-reactivity detected for other porcine viruses. NC, negative control without templates. ** *p* < 0.01 by Student *t* test.

To validate the specificity of the CRISPR-LbCas12a/LbuCas13a detection systems, we tested eight other porcine viruses, including porcine circovirus type 2 (PCV-2), PCV-3, porcine kobuvirus (PKV), pseudorabies virus (PRV), porcine epidemic diarrhea virus (PEDV), porcine reproductive and respiratory syndrome virus (PRRSV), classical swine fever virus (CSFV), and porcine parvovirus (PPV) (Figure 6). Fluorescence and lateral flow strip (LFS) analysis demonstrated that the both CRISPR assays exclusively detected ASFV, with no cross-reactivity observed against the other eight pig viruses (Figure 6A–D).

### 3.5. Detection of Clinical Samples by RPA-CRISPR-LbCas12a/LbuCas13a Systems Using Column Purified DNA

**Figure 7:**
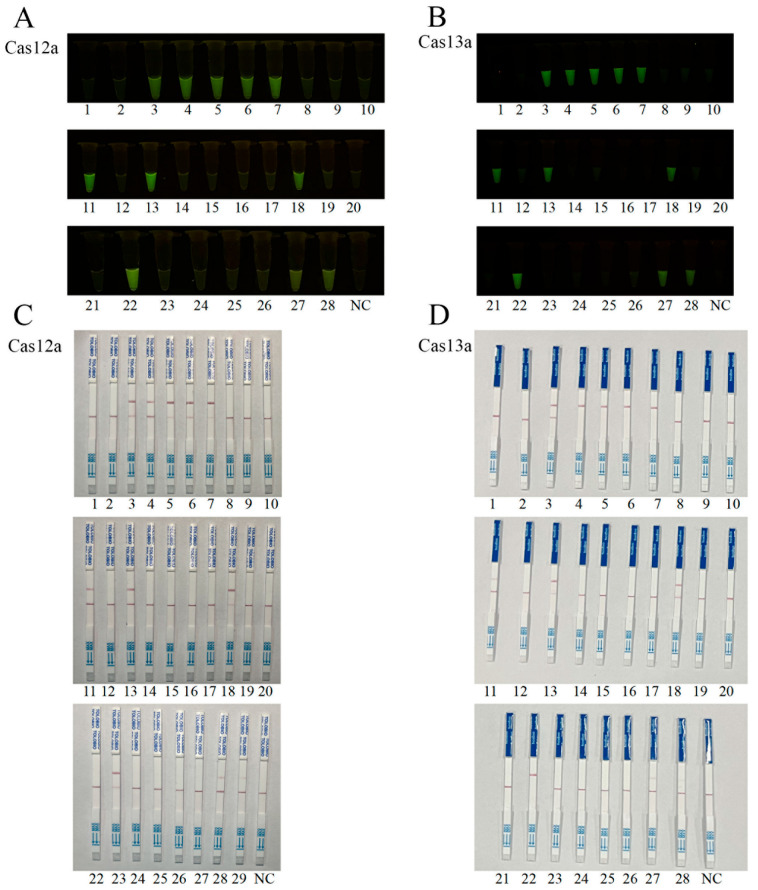
Clinical sample detections using CRISPR-LbCas12a/LbuCas13a with DNA extracted and purified by column-based method: (**A**,**B**) CRISPR-LbCas12a (**A**) and CRISPR/LbuCas13a (**B**) fluorescence detections of 28 clinical specimens. Positive samples: Nos. 3, 4, 5, 6, 7, 11, 13, 18, 22, 27, and 28, in total 11 samples. Negative samples: Nos. 1, 2, 8–10, 12, 14–17, 19–21, 23–26, in total 17 samples. Results from LbCas12a and LbuCas13a detections were 100% concordant. (**C**,**D**) CRISPR-LbCas12a-LFS (**C**) and LbuCas13a-LFS (**D**) detections of the same 28 samples, showing complete agreement with fluorescence results. NC, negative controls of water.

To evaluate the clinical utility of the CRISPR-LbCas12a/LbuCas13a detection systems, genomic DNA was extracted and purified from 28 clinical tissue samples using a column-based method (HiPure Tissue DNA Mini Kit), and subjected to parallel detections by both CRISPR-Cas assays. Both CRISPR detections identified the same 11 ASFV-positive out of the 28 samples (Figure 7A,B). Fluorescence and lateral flow strip (LFS) readouts in both CRISPR detections were 100% concordant (Figure 7A–D). All 28 samples were also subjected to quantitative PCR (qPCR) following the WOAH (World Organization for Animal Health) recommended protocol, and the qPCR results exhibited complete agreement with CRISPR-Cas12a/Cas13a detection outcomes (Table 1).

### 3.6. Detection of Clinical Samples by RPA-CRISPR-LbCas12a/LbuCas13a Systems Using Lysis-Extracted DNA

**Figure 8:**
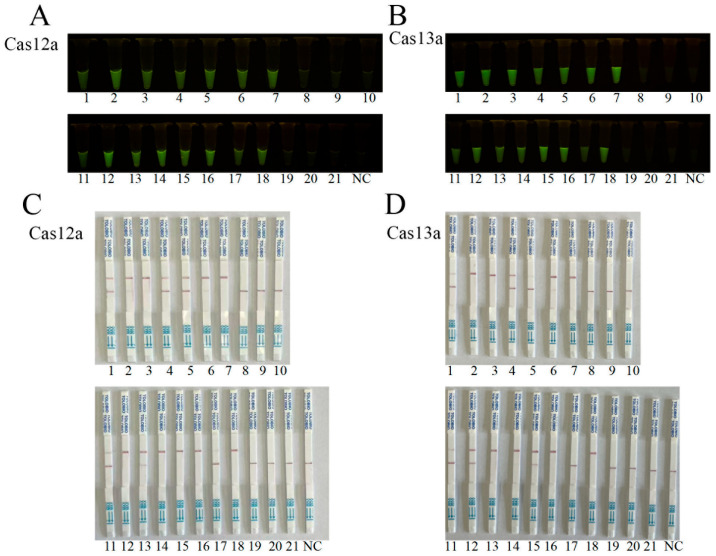
Clinical sample detections using CRISPR-LbCas12a/LbuCas13a with rapid lysis-extracted DNA: (**A**,**B**) CRISPR-LbCas12a (**A**) and CRISPR/LbuCas13a (**B**) fluorescence detections of 21 clinical specimens. Positive samples: Nos. 1–7 and 11–18, in total 15 samples. Negative samples: Nos. 8–10 and 19–21, in total 6 samples. Results from LbCas12a and LbuCas13a detections were 100% concordant. (**C**,**D**) CRISPR-LbCas12a-LFS (**C**) and LbuCas13a-LFS (**D**) detections of the same 21 samples. With LbCas12a-LFS 15 positive and LbuCas13a-LFS 14 positive. NC, negative controls of water.

To evaluate the applicability of a rapid lysis-based DNA extraction method, we analyzed 21 clinical specimens using the CRISPR-LbCas12a/LbuCas13a detection systems with the lysis-extracted DNA. The results identified 15 ASFV-positive out of the 21 samples based on the fluorescence signals obtained in CRISPR-LbCas12a and CRISPR-LbuCas13a detection systems, which exhibited complete concordance (Figure 8A,B). As for the LFS detection, LbCas12a-LFS detected the same 15 positive samples as in the fluorescence signals, whereas LbuCas13a-LFS detected 14 positives out of the 21 samples (Figure 8C,D). The qPCR analysis confirmed the 15 positives from 21 clinical samples (Table 2). These findings demonstrated that the lysis-based DNA extraction method is highly compatible with CRISPR-Cas systems for ASFV detection in clinical samples, offering a rapid (<15 min), equipment-free alternative to column-based DNA extraction and purification.

## 4. Discussion

Since its introduction to the Caucasus region in 2007, ASFV has progressively spread to Central Europe and Russia [1,27]. With its first emergence in China in 2018, ASFV has caused significant losses to the swine industry [27]. Given the unavailability of licensed vaccines, ASFV prevention and control mainly depend on early diagnosis and culling measures [2,4]. Early detection, reporting, and intervention are essential for effective containment and eradication, necessitating rapid, field-deployable diagnostic tools to facilitate timely epidemiological control [7,8]. The RPA-CRISPR platform is characterized by minimal technical expertise requirements, short reaction time, and suitability for on-site testing [9,28]. Previously, we established the RPA-CRISPR-LbCas12a and RPA-CRISPR-LwCas13a detections, separately, based on the ASFV structure gene p17/D117L [25,29]. Here, we leveraged the RPA-CRISPR-LbCas12a and RPA-CRISPR-LwCas13a systems to develop parallel and visual ASFV detections.

ASFV pS273R, the sole cysteine protease encoded by the ASFV genome, facilitates viral replication by modulating host immune responses [18,20,22,23]. The encoding gene S273R is highly conserved across different genotypes of ASFV strains (Tables S2 and S3). Here, we established a dual-modal fluorescence and lateral flow strip (LFS) assay targeting the S273R gene, integrating RPA amplification with CRISPR-LbCas12a/LbuCas13a-mediated detection. Multiple crRNAs were designed and screened for optimal performance. Furthermore, systematic optimization of reaction parameters, including crRNA concentration, Cas protein dosage, and probe concentration, yielded the refined detection systems. Coupled with RPA amplification, it demonstrated that the RPA-CRISPR-LbCas12a/LbuCas13a detection systems have a sensitivity of 2 copies/μL of the S273R gene in both fluorescence and LFS readouts. Specificity testing against the other eight porcine viruses revealed no cross-reactivity.

For clinical sample detections, two nucleic acid extraction methods, column-based extraction and rapid lysis-based DNA extraction, were evaluated. Among the 28 clinical samples processed via column-based extraction and purification, the RPA-CRISPR-LbCas12a/LbuCas13a assays exhibited 100% concordance with qPCR results. For the 21 samples analyzed using rapid DNA lysis, the RPA-CRISPR-LbCas12a/LbuCas13a assays achieved more than a 95.45% concordance rate with qPCR. These findings underscore the reliability of the RPA-CRISPR-LbCas12a/LbuCas13a platform for field-deployable ASFV detection, offering a robust tool to mitigate ASF outbreak impacts through early diagnosis. The lower quality of DNA from field samples is a common problem for all detection methods. Our RPA-CRISPR-LbCas1a/LbuCas13a detection methods achieved a high sensitivity, detecting as low as two copies of the S273R gene; they are particularly useful in the detection of field samples by counteracting the lower quality of DNA.

The parallel RPA-CRISPR-LbCas1a/LbuCas13a detection methods established in our study will significantly enhance the feasibility of on-site ASFV detection. However, due to the risk of aerosol contamination from RPA, which may lead to false-positive results, further investigation is required to devise amplification-free CRISPR/Cas-based approaches [30,31]. Such an advancement will fulfill a convenient and reliable on-site detection for clinical applications.

## Figures and Tables

**Table 1:** Comparison of the detection results between RPA-LbCas12a/LbuCas13a (with column-extracted and purified DNA) and qPCR.

Sample Types	Numbers of Samples	Fluorescence Results		LFS Results		qPCR Results
		LbCas12a	LbuCas13a	LbCas12a	LbuCas13a	
Heart	4	1/3	1/3	1/3	1/3	1/3
Liver	5	2/3	2/3	2/3	2/3	2/3
Spleen	4	1/3	1/3	1/3	1/3	1/3
Lung	7	4/3	4/3	4/3	4/3	4/3
Kidney	2	0/2	0/2	0/2	0/2	0/2
Intestine	2	1/1	1/1	1/1	1/1	1/1
Lymph node	2	0/2	0/2	0/2	0/2	0/2
Oral swab	2	2/2	2/2	2/2	2/2	2/2
Total	28	11/17	11/17	11/17	11/17	11/17
Positive rate	/	39.29%	39.29%	39.29%	39.29%	39.29%

Note: the x/y denotes number of positive/number of negative.

**Table 2:** Comparison of detection results between RPA-LbCas12a/LbuCas13a (with lysis-extracted DNA) and qPCR.

Sample Types	Number of Samples	Fluorescence Results		LFS Results		qPCR Results
		LbCas12a	LbuCas13a	LbCas12a	LbuCas13a	
Heart	2	1/1	1/1	1/1	1/1	1/1
Liver	3	3/0	3/0	3/0	3/0	3/0
Spleen	6	4/2	4/2	4/2	4/2	4/2
Lung	7	5/2	5/2	5/2	4/3	5/2
Intestine	2	1/1	1/1	1/1	1/1	1/1
Oral swab	1	1/0	1/0	1/0	1/0	1/0
Total	21	15/6	15/6	15/6	14/7	15/6
Positive rate	/	71.42%	71.42%	71.42%	66.67%	71.42%

Note: the x/y denotes number of positive/number of negative.

## Data Availability

The authors confirm that the data supporting the findings of this study are available within the article and its Supplementary Materials.

## Supplementary Materials

The following supporting information can be downloaded at: https://www.mdpi.com/article/10.3390/ani15131902/s1. Supplementary Table S1. Primers used in this study. Table S2. Sequence identity of Cas12a-RPA-S273R-1 and Cas12a-S273R-crRNA1 with the S273R sequences across different ASFV strains. Table S3. Sequence identity of Cas13a-RPA-S273R-3 and Cas13a-S273R-crRNA1 with the S273R sequences across diverse ASFV strains.
