# Supplementary material for: Parallel and Visual Detections of ASFV by CRISPR-Cas12a and CRISPR-Cas13a Systems Targeting the Viral S273R Gene
Source: Animals (Basel). 2025 Jun 27;15(13):1902. doi: 10.3390/ani15131902 (PMC12248759; doi:10.3390/ani15131902)
Supplement: Supplementary file 1 [file animals-15-01902-s001.zip › Table S1.pdf]

Supplementary Table S1. Primers used in this study

Primer names		Sequences (5' - 3')
Cas12a-T-S273R	F:	AATGTCTATATTAGAAAAAATTACGTCAAGTCCCTCTGAATGCGCAGAGC
	R:	GACGAAGACATGTATAAGTTTAGAACCCATCTGTTTCGCATCGCATAAAC
Cas12a-S273R-crRNA1	F:	GAAATTAATACGACTCACTATAGGAATTTCTACTAAGTGTAGATTGGATATGCGGGGCGACTGCTGG
	R:	CCAGCAGTCGCCCCGCATATCCAATCTACACTTAGTAGAAATTCCTATAGTGAGTCGTATTAATTTC
Cas12a-S273R-crRNA2	F:	GAAATTAATACGACTCACTATAGGAATTTCTACTAAGTGTAGATAATCAAGTGGATATGGTAAAGGTA
	R:	TACCTTTACCATATCCACTTGATTATCTACACTTAGTAGAAATTCCTATAGTGAGTCGTATTAATTTC
Cas12a-S273R-crRNA3	F:	GAAATTAATACGACTCACTATAGGAATTTCTACTAAGTGTAGATCCTTCTACATATTGTAGCTCTTC
	R:	GAAGAGCTACAATATGTAGAAGGATCTACACTTAGTAGAAATTCCTATAGTGAGTCGTATTAATTTC
Cas12a-RPA-S273R-1	F:	GCGTTTTAAACACGGACTTTTCAACGGGCACTGG
	R:	CTGCAAGCGTTTTCACGGTGTGGTGTATTTTTA
Cas12a-RPA-S273R-2	F:	ACTTTTCAACGGGCACTGGAAAACACTGGGTAGCC
	R:	TTGGTAACTGCAAGCGTTTTCACGGTGTGGTGTA
Cas13a-T-S273R	F:	GAAATAATACGACTCACTATAGGGATGTCTATATTAGAAAAAATTACGTCAAGTCC
	R:	TGCGATGCGAAACAGATGGGT
Cas13a-S273R-crRNA1	F:	GAAATTAATACGACTCACTATAGGGGACCACCCCAAAAATGAAGGGGACTAAAACTCAATATTGAAGTTTGTAAGAAGACCCG
	R:	CGGGTCTTCTTACAAACTTCAATATTGAGTTTTAGTCCCCTTCATTTTTGGGGTGGTCCCCTATAGTGAGTCGTATTAATTTC
Cas13a-S273R-crRNA2	F:	GAAATTAATACGACTCACTATAGGGGACCACCCCAAAAATGAAGGGGACTAAAACTTAAACCGAGTCTCCAGTTCTTTGGAGA
	R:	TCTCCAAAGAACTGGAGACTCGGTTTAAGTTTTAGTCCCCTTCATTTTTGGGGTGGTCCCCTATAGTGAGTCGTATTAATTTC
Cas13a-RPA-S273R-1	F:	GAAATTAATACGACTCACTATAGGGCACTCGGTTGCGATTCGGAGTCCTGCGTAATTAC
	R:	TCATGGAAAAAGGACAGTTGAAAAACTTGGTGTA
Cas13a-RPA-S273R-2	F:	GAAATTAATACGACTCACTATAGGGCCTGCGTAATTACCCACCCCGCCGTGAAGGCCTA
	R:	ATTTATAATGGACCCTCTCAAAGTCCATCATGGA
Cas13a-RPA-S273R-3	F:	GAATTAATACGACTCACTATAGGGAAAGAGACACTCGGTTGCGATTCGGAGTCC
	R:	TCAAAGTCCATCATGGAAAAAGGACAGTTG

Cas12a-FQ-Probe	6-FAM-CCGGAAAAAAAAAAAACCGG-BHQ
Cas12a-Biotin-Probe	6-FAM-TTTTTTTATTTTTTT-Biotin
Cas13a-FQ-Probe	6-FAM-rUrUrUrUrArUrU-BHQ
Cas13a-Biotin-Probe	6-FAM-UUUUUUUUAUUUUUU-Biotin

Note: Cas12a-T-S273R primers are used for PCR amplification of template DNA from pCAGGS-S273R (Figure 2A), whereas Cas13a-T-S273R primers are used for PCR amplification of DNA from pCAGGS-S273R which then transcribed by T7 RNA polymerase into template RNA (Figure 2D).

The primers of Cas12a-RPA-S273R-1 was determined as the best RPA primers (Figure 5A) for subsequent RPA-CRISPR-Cas12a detection, whereas the primers of Cas13a-RPA-S273R-3 was determined as the best RPA primers (Figure 5B) for subsequent RPA-CRISPR-Cas13a detection. F, forward primers; R, reverse primers.
